# Reduced strength and increased variability of extinction selectivity during mass extinctions

**DOI:** 10.1098/rsos.230795

**Published:** 2023-09-27

**Authors:** Pedro M. Monarrez, Noel A. Heim, Jonathan L. Payne

**Affiliations:** ^1^ Department of Earth and Planetary Sciences, Stanford University, Stanford, CA 94305, USA; ^2^ Department of Earth and Climate Sciences, Tufts University, Medford, MA 02155, USA

**Keywords:** extinction selectivity, body size, geographical range, mass extinctions, Phanerozoic

## Abstract

Two of the traits most often observed to correlate with extinction risk in marine animals are geographical range and body size. However, the relative effects of these two traits on extinction risk have not been investigated systematically for either background times or during mass extinctions. To close this knowledge gap, we measure and compare extinction selectivity of geographical range and body size of genera within five classes of benthic marine animals across the Phanerozoic using capture–mark–recapture models. During background intervals, narrow geographical range is strongly associated with greater extinction probability, whereas smaller body size is more weakly associated with greater extinction probability. During mass extinctions, the association between geographical range and extinction probability is reduced in every class and fully eliminated in some, whereas the association between body size and extinction probability varies in strength and direction across classes. While geographical range is universally the stronger predictor of survival during background intervals, variation among classes during mass extinction suggests a fundamental shift in extinction processes during these global catastrophes.

## 1. Introduction

**Figure 1.**
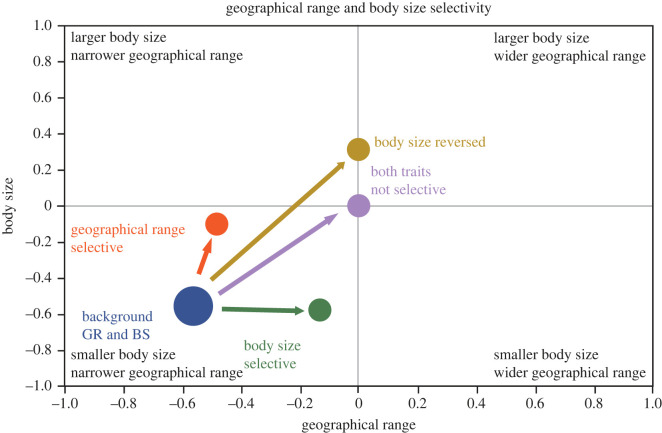
Conceptual bivariate representation of body size and geographical range extinction selectivity during background intervals and mass extinctions. Based on previous analyses, narrower geographical range and smaller body size are selective for extinction during background intervals [10,12,13]. During mass extinctions body size is either selective in the same direction as background extinction, not selective at all, or reverses direction of selectivity [10]. Mass extinction geographical range selectivity is in the same direction as background extinction or is not selective but does not reverse [1,12].

A central debate in evolutionary biology focuses on whether extinction selectivity during mass extinction events differs from that during background times [1–3]. Previous work has demonstrated that various traits are associated with extinction probability during background intervals and mass extinction events [4–9]. Furthermore, extinction selectivity with respect to body size changes strength and, in some cases, direction between background and mass extinction [10]. The same may apply for geographical range and other ecological traits [1,11,12]. Despite studies demonstrating varying degrees of selectivity for different traits during background and mass extinction, it is unclear which of these traits is more determinative of extinction versus survival and whether that also changes between extinction regimes. The strength of selectivity could change such that one trait is selected upon more strongly during background intervals and another is the stronger determinant of survivorship during mass extinction (figure 1). Alternatively, one trait could remain the more important determinant of survivorship under both regimes even if extinction selectivity differs significantly between background and mass extinction for two (or more) different traits. This question remains unanswered, even for the most-hypothesized determinants of extinction, because large databases of fossil body size and fossil geographical occurrences have only recently been compiled, and the computational power to conduct these analyses has only become recently available [14,15]. Few studies have explicitly examined extinction selectivity of multiple traits simultaneously between background intervals and mass extinction events (but see [14,16–18]), and differences in datasets further impede comparisons across studies. Because stronger selectivity imparts greater effect on the surviving biota at any given extinction intensity [15], quantifying selectivity on a common scale for multiple traits is a necessary step in demonstrating how extinction selectivity shapes the global biota over time and in extrapolating patterns from the fossil record to predict consequences of current and future biodiversity crises [16].

Geographical range and body size are two of the traits most commonly hypothesized to influence extinction risk and most used to test hypothesized extinction selectivity in the fossil record. Broad geographical range is interpreted to reduce vulnerability to extinction by enabling species and higher taxa to find refuge from local to regional environmental and ecological stresses [1,6,7,12,19–21]. Body size scales allometrically with various important traits, such as trophic level, fecundity, generation time and total metabolic rate [22–25]. Consequently, body size has long been hypothesized and demonstrated to correlate with extinction risk under a wide range of extinction scenarios [13,22,26–35].

Growing evidence suggests that extinction selectivity differs between background intervals and mass extinctions events. Most notably, selectivity for geographical range [1,12,36], biogeography [11] and body size [10] all differ in magnitude, direction or both between background intervals and mass extinction events. The relationship between geographical range and extinction is strongest during background intervals [1,12,36]. During mass extinction intervals, however, geographical range selectivity is weaker or absent [1,12,33]. Extinction is also selective with respect to body size during background intervals, with smaller marine animals exhibiting greater extinction risk in many classes [13]. By contrast, selectivity with respect to body size varies by clade during mass extinction events [10]. What remains unclear, however, is whether extinction is more selective on body size or geographical range under each extinction regime and, if so, by how much. In other words, does the alternation of macroevolutionary regimes cause a shift in the identity of the most important determinant of extinction?

Previous studies testing for changes in extinction selectivity of geographical range between background and mass extinction events, however, have primarily focused on select higher taxa and on individual extinction events [1,7,36], or spanned the Phanerozoic but used data aggregated across multiple, distantly related phyla [12]. Additionally, these studies do not formally test for alternating macroevolutionary regimes, nor consider sampling completeness in estimating extinction selectivity, which can distort apparent extinction patterns under some circumstances [37–39]. To our knowledge, Monarrez *et al*. [10] is the only study that directly and explicitly tests for changes in macroevolutionary regimes across the Phanerozoic while also considering sampling completeness in estimating selectivity on body size.

Here, we assess and compare extinction selectivity with respect to geographical range and body size within a single dataset to determine which of these traits is more determinative of survivorship during different macroevolutionary regimes. We test for changes in macroevolutionary regimes in fossil marine animals between background intervals and the canonical ‘Big Five’ mass extinction events [40] using the marine animal fossil record spanning from 485 to 1 Ma and considering geographical range and body size as predictors of extinction. We quantify extinction selectivity and model the influence of temporal variation in sampling completeness and body-size bias in sampling using capture–mark–recapture (CMR) models and determine model weights using the Akaike information criterion corrected for sample size (AICc). Moreover, because geographical range scales with body size in various taxonomic groups (e.g. [25,41–44]) we also test for a possible interactive effect between geographical range and body size in extinction selectivity.

## 2. Material and methods

For this study, we use a genus-level dataset of fossil marine animal body sizes and geographical ranges, focusing on benthic, solitary bilaterians [45]. Body size is measured as the log_10_ transformation of biovolume (mm^3^) calculated from measurements made on figured specimens from primary literature [45]. Geographical range is measured as the log_10_ transformation of the maximum great circle distance (km) of each genus in each time interval analysed. Only classes with a minimum of 500 genera with body size measurements and stage-resolved stratigraphic ranges were used for this study. The analysed dataset comprises 6562 genera belonging to five classes: Bivalvia (1603), Gastropoda (1767), Rhynchonellata (1827), Strophomenata (823) and Trilobita (583).

To quantify extinction selectivity, we matched our body-size data with corresponding stage-resolved genus occurrences from the Paleobiology Database [46], downloaded on 24 October 2022. We treated the occurrence data file by first removing genera for which we do not have corresponding data for body size, those without any stage-resolved occurrences, and we removed occurrences from remaining genera that were not resolved to a single stage. As is conventional in analyses of Phanerozoic diversity patterns, subgenera were elevated to genus level [47,48]. We omitted Cambrian occurrence data for this study because Cambrian stratigraphic range resolution in the Paleobiology Database is not well constrained at the stage level. Occurrences from this file were also used to calculate the maximum great circle distance between occurrences for each genus in each stage as a time-varying measure of geographical range using the R package *fossil* [49]. Maximum great circle distance reliably estimates the geographical range of a taxon even during time intervals with limited fossil occurrences [50]. Stages within the stratigraphic range of a genus for which the genus lacked any or only had a single occurrence were assigned a geographical range of 1 km prior to log-transforming the dataset. We take this approach because it assigns a smaller range than that for higher sampled genera, which is appropriate given that ranges are based on sampling patterns. Because body size and geographical range are measured in different units (mm^3^ and km, respectively) and have separate distributions, we calculated a standard score (i.e. adjusted the distribution to have a mean of zero and a standard deviation of one) by class for body size across the whole dataset (because only a single size is assigned for each genus) and for geographical range by class and time interval (because range is assigned to each genus on a stage-by-stage basis) before quantifying extinction selectivity. Thus, the coefficients from the CMR analysis reflect the effect of a one-standard-deviation change in the predictor variable on the log-odds of extinction (ln(*q*/(1 − *q*)), where *q* is the probability of extinction). The final occurrence file consists of 242 871 total occurrences, with 88 856 Bivalvia, 53 673 Gastropoda, 57 297 Rhynchonellata, 33 935 Strophomenata and 9110 Trilobita occurrences.

To estimate the association of body size and geographical range with probability of extinction, we applied a CMR framework to estimate extinction probability as a function of body size, geographical range and time interval across geologic stages using a combination of additive and multiplicative models following Monarrez *et al*. [10]. CMR estimates the number of taxa (genera in this case) that are initially observed in a given time bin (capture and mark) and estimates the number of genera from the initial capture that survive into subsequent time bins (recapture). This estimation is done by calculating the probability that each genus survives between time bins by conditioning on the number of genera that were observed in the initial time bin while calculating the probability that a genus is recaptured assuming it survived between time bins. This is mathematically expressed as

2.1 $$S_{t+1}=\phi_{t}+\frac{1}{p_{t+1}},$$

where *S_t_*_+1_ is the estimate of surviving genera in a given time bin (*t* + 1) conditioned on the number of genera observed in the initial time bin (*t*), *ϕ_t_*_+1_ is the probability of each genus surviving between time bins following the initial time bin, and *p_t_*_+1_ is the probability that a genus was sampled in a given time bin if it survived between time bins following the initial time bin [51]. To estimate the probability of extinction, the complement of the estimate of surviving genera is taken, and is expressed as

2.2 $$E_{t}=1-\left(\frac{S_{t+1}}{s_{t}}\right),$$

where *E_t_* is the probability of extinction in a time bin, and *s_t_* is the total number of genera observed in that time bin [51]. The probability of extinction for each genus is calculated for each geologic stage, and it is the dependent variable in a logistic regression formula that takes the form:

2.3 $$E_{p}∼\mathrm{time}+\mathrm{trait},$$

where *E_p_* is the probability of extinction for each genus for each time bin, time is each geologic stage, and trait is geographical range and or body size. This approach assumes that each geologic interval is equivalent and is used to calculate extinction selectivity during background intervals. To calculate extinction selectivity for mass extinction events, we coded each time interval as being background (0) or mass extinction (1) and included mass extinction as an interaction term in our logistic regression formulae, taking the form

2.4 $$E_{p}∼\mathrm{time}+\mathrm{trait}\times \mathrm{mass}\;\mathrm{extinction}.$$

There are multiple CMR model frameworks that can be used based on the research question addressed. Here we use the Pradel Seniority model [52], which differs from traditional CMR models that are conditioned to estimate survivorship on the first occurrence of a taxon, and instead estimates survivorship and recruitment unconditionally based on the number of time bins. Because the Pradel Seniority model includes estimates of recruitment (which can be used to estimate origination in the fossil record), it substantially increases the number of individual models for each genus, totaling 121 for this study (the full list of models can be found in electronic supplementary material, table S1). This approach has primarily been applied to ecological studies, but it has recently been applied to palaeontological data and it can be used to estimate extinction and origination rates, selectivity and taxonomic richness [10,13,53–56].

Monarrez *et al*. [10] demonstrated that the definition of mass extinction events does not meaningfully affect overall results of body size selectivity, and it also does not affect overall results of geographical range selectivity (electronic supplementary material, figure S1). Thus, we focused on the canonical ‘Big Five’ mass extinction events for this analysis. For each class, we compared support for models assuming distinct extinction selectivity regimes between background and mass extinction with models assuming a single selectivity regime across the entire study interval. Best-fit models were identified using Aikake information criterion scores corrected for sample size (AICc) and associated model weights [57]. The coefficients of association between body size, geographical range and extinction from models that garnered at least 0.01 weight were averaged across all models and were used to quantify selectivity regimes for comparison of selectivity strength and direction among classes between background and mass extinctions.

In sum, we fitted 605 models on our five classes (121 models per class) using time interval, body size, geographical range and mass extinction (or associated recovery interval) as independent predictors of extinction (or origination) probabilities and using body size and time interval for estimating sampling probabilities. Although origination is included within our CMR models, we do not consider the role of origination in this analysis following the approach of [53]. We performed our CMR analysis using the R package *RMark* [58] which is the R interface for the Mark program [59]. Given the computationally expensive nature of these CMR analyses, we used the Sherlock high-performance computing cluster managed by the Stanford Research Computing Center at Stanford University. All analyses were conducted using R version 4.0.2 [60].

## 3. Results

Models with distinct selectivity regimes garner the greatest support for three of the five classes, with each of these models containing both body size and geographical range as covariates (table 1). The only classes for which a model with a single selectivity regime garnered the greatest support are the brachiopod classes Rhynchonellata (0.47) and Strophomenata (0.44). Of the three classes that garner the greatest support for a distinct selectivity regime model (Bivalvia, Gastropoda and Trilobita), only gastropods garner the greatest support for a model with both body size and geographical range (0.87). Bivalves receive the greatest model support for distinct selectivity regimes for geographical range (0.57), whereas trilobites receive the greatest model support for distinct selectivity regimes for body size (0.57). Moreover, only rhynchonellate brachiopods received the greatest support for a model with an interaction term between geographical range and body size, whereas every other class lacked support. Therefore, we omit standalone selectivity results for models with the geographical range and body size interaction.

**Table 1.** CMR extinction model selection table with the top four models for each class. Origination is included in extinction models but is not considered for this analysis. Models with only time, body size (BS) and/or geographical range (GR) covariates correspond to a single regime model. Models with interactions with mass extinction (ME) or recovery (Rec) covariates correspond to two-regime models. For the full table, see electronic supplementary material, table S1.

class	rank	extinction	origination	sampling	*Δ*AICc	weight
Bivalvia	1	∼time + BS + GR × ME	∼time + BS × Rec + GR × Rec	∼time + BS	0	0.57
	2	∼time + BS + GR × ME	∼time + BS × GR × Rec	∼time + BS	1.49	0.27
	3	∼time + BS × GR × ME	∼time + BS × GR × Rec	∼time + BS	2.61	0.16
	4	∼time + BS × GR × ME	∼time + BS × Rec + GR	∼time + BS	17.61	0
Gastropoda	1	∼time + BS × ME + GR × ME	∼time + BS × GR × Rec	∼time + BS	0	0.87
	2	∼time + size × GR × ME	∼time + BS × GR × Rec	∼time + BS	5.01	0.07
	3	∼time + BS × GR	∼time + BS × GR × Rec	∼time + BS	6.55	0.03
	4	∼time + BS × ME + GR × ME	∼time + BS × Rec + GR	∼time + BS	8.33	0.01
Rhynchonellata	1	∼time + BS × GR	∼time + BS × Rec + GR	∼time + BS	0	0.47
	2	∼time + BS × GR	∼time + BS × Rec + GR × Rec	∼time + BS	0.9	0.3
	3	∼time + BS × GR × ME	∼time + BS × Rec + GR	∼time + BS	1.82	0.19
	4	∼time + BS × GR	∼time + BS × GR × Rec	∼time + BS	4.74	0.04
Strophomenata	1	∼time + GR	∼time + BS × Rec + GR	∼time + BS	0	0.44
	2	∼time + BS × GR	∼time + BS × Rec + GR	∼time + BS	1.71	0.19
	3	∼time + GR	∼time + BS × Rec + GR × Rec	∼time + BS	2.12	0.15
	4	∼time + BS × GR	∼time + BS × Rec + GR × Rec	∼time + BS	3.86	0.06
Trilobita	1	∼time + BS × ME + GR	∼1	∼time + BS	0	0.57
	2	∼time + BS × ME + GR × ME	∼1	∼time + BS	1.21	0.31
	3	∼time + BS × GR × ME	∼1	∼time + BS	4.45	0.06
	4	∼time + BS + GR × ME	∼1	∼time + BS	6.47	0.02

**Figure 2.**
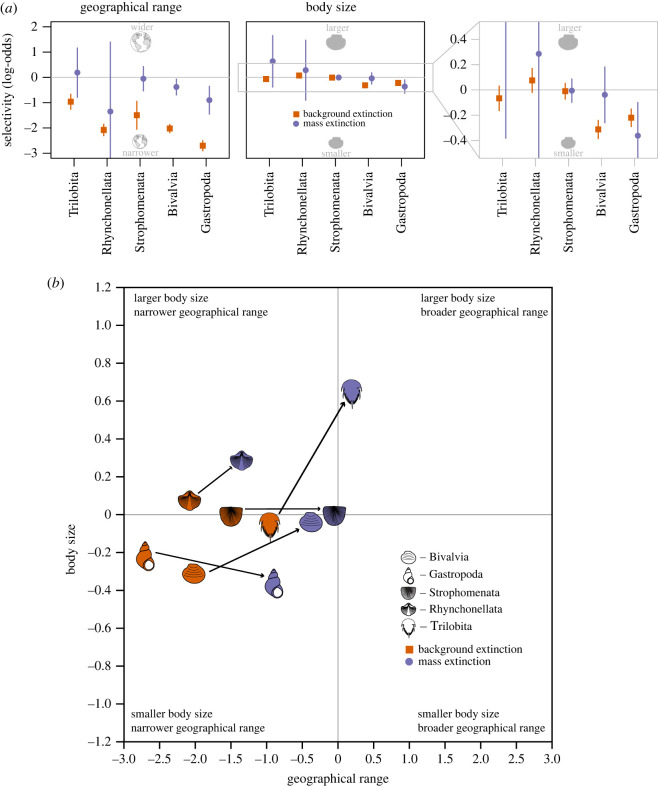
Relationship of extinction risk of marine animal genera by class by geographical range and body size. (*a*) Model-averaged logistic regression coefficients from the CMR results indicate the log-odds estimate of extinction as a function of geographical range and body size. Coefficients represent the average extinction risk across all background and mass extinction intervals considered in this study. Changes in the direction and magnitude of coefficients during mass extinctions relative to background intervals indicate alternating selectivity regimes for both geographical range and body size. Plot on the right is the enlarged inset box. The confidence intervals for some points are smaller than plot symbols. (*b*) Bivariate plot of logistic regression coefficient estimates from (*a*) for each class during background intervals (orange) and mass extinctions (lavender). Selectivity during mass extinction tends to move closer to zero as indicated by the arrows, demonstrating that selectivity reduces and becomes more variable in general across most classes during mass extinctions relative to background intervals. Trilobite, bivalve and gastropod graphics modified from PhyloPic, which are publicly available under Creative Commons licences.

To quantify the direction and magnitude of selectivity during background intervals and mass extinction, we use the logistic regression coefficients from the CMR models. Because each class has several models that receive support greater than 0.01 (29 models from the total of 605; for full table, see electronic supplementary material, table S1), we averaged the coefficients of all the models with weight greater than or equal to 0.01 for each class, weighted by proportional AICc model support, and used them to quantify extinction selectivity. Geographical range coefficients indicate that narrow geographical range is strongly associated with increased extinction risk for all five classes during background intervals (figure 2*a*). During mass extinctions, however, extinction selectivity with respect to geographical range decreased substantially, with results indicating no significant selectivity for Trilobita, Rhynchonellata and Strophomenata, and reduced selectivity strength for Bivalvia and Gastropoda. Whereas selectivity is only significant for bivalves and gastropods during background intervals, selectivity with respect to body size tends to be weaker than that with respect to geographical range for all classes. Trilobites, strophomenate brachiopods, bivalves and gastropods exhibit a tendency towards extinction of smaller-bodied genera, whereas rhynchonellate brachiopods demonstrate slight selectivity against larger-bodied genera; however, only bivalves and gastropods exhibit significant selectivity (figure 2*a*). During mass extinctions, selectivity with respect to body size varies by class. Trilobites show stronger selectivity against larger body size, gastropods show selectivity against smaller body size, whereas both brachiopod classes and bivalves lack measurable selectivity. Despite the reduction of geographical range selectivity across all classes during mass extinction, geographical range varies by class such that for some classes it is not selective, but it is for others. Moreover, whereas geographical range is more selective during background extinction, geographical range selectivity is reduced enough during mass extinction such that body size might be more selective for trilobites (figure 2).

## 4. Discussion

Across all classes, extinction selectivity with respect to geographical range is substantially stronger than for body size, particularly during background intervals, further supporting the importance of geographical range in macroevolutionary dynamics [4,7]. Body size is also important during background intervals within some classes; however, geographical range selectivity is so pervasive that body size is of lower consequence at the clade level over most of the Phanerozoic (figure 2). Geographical range selectivity within classes shows a similar change between background intervals and mass extinctions to that observed when all benthic taxa are combined [12], even after controlling for temporal variation in sampling completeness and size-based sampling bias. Despite classes exhibiting selectivity with respect to geographical range and/or body size during mass extinction, the results of this study generally indicate that mass extinctions might be less selective than background intervals (figure 2*b*). This reduction in selectivity during mass extinctions is driven primarily by reduced geographical range selectivity because it is so strongly selective during background intervals. Moreover, selectivity during background intervals tends to be largely shared, even if not all selectivity coefficients are statistically significant, for both geographical range and body size, whereas selectivity varies more substantially by clades during mass extinctions.

Many studies have demonstrated that selectivity is reduced during mass extinctions or that they are non-selective relative to background intervals [10,12,36,61–67]. Similarly, previous studies have documented varying responses to extinction events by taxonomic group based on different traits [11,14,16,17,68], but not variable responses by clades using the same trait. To our knowledge, no previous study has documented a reduction of selectivity or a change from shared to variable selectivity for the same trait across multiple disparate clades during mass extinctions. This gap could stem from the recent development of large datasets with traits that can be parsed out by clades and span most of the Phanerozoic combined with the computational power to measure selectivity while considering sampling completeness. Nevertheless, the results of this study suggest that selectivity during mass extinctions is driven primarily by differing interactions between intrinsic traits with extrinsic events among clades and could make predicting mass extinction selectivity in the marine biosphere more difficult than previously realized [69].

The increased variability of selectivity across clades during mass extinctions relative to background intervals suggests that mass extinctions briefly alter fundamental macroevolutionary dynamics at the class level. These observations, however, could also be potentially explained by how selectivity is averaged across temporal bins in our CMR models. Because our analysis is conducted at the stage level, there is a disproportionate number of background intervals (81) relative to mass extinction intervals (5). As such, the minimal variation of selectivity observed during background intervals could result from regression coefficients reflecting an average of patterns across many more background intervals than mass extinctions. This would be a problem of particular importance if uncertainty around model coefficient estimates results from violation of the assumption intrinsic to the models that mass extinctions share selectivity patterns. Whereas there is overlap between background and mass extinction coefficients because of the greater uncertainty in selectivity during mass extinctions, models with separate background and mass extinction selectivity are best supported for most classes. Nevertheless, the direction of selectivity during mass extinctions observed in this study is consistent with previous studies that measure selectivity of specific clades across different mass extinction events, particularly bivalves [1,36,70–72], gastropods [1] and rhynchonellates [73].

Geographical range extinction selectivity during background intervals is consistent with previous analyses [1,7,12]. Genera with narrower geographical ranges are more vulnerable to extinction from background processes, such as biotic interactions, narrow functional niches and local to regional changes in environmental conditions. It would be expected that geographical range selectivity should decrease during mass extinction events, where global scale environmental perturbations exert selective stresses more evenly, and a wider geographical range fails to buffer genera from extinction, which is largely observed here. However, the molluscan classes in this study still exhibit preferential extinction of narrowly ranging genera during mass extinctions, albeit reduced relative to background intervals. These results are consistent with previous studies documenting geographical range selectivity during the End-Cretaceous mass extinction, particularly for bivalves and gastropods [36,74]. While taxonomic data were not parsed by clade, Payne & Finnegan [12] also found a significant association between geographical range and survivorship, albeit small relative to background intervals during the End-Cretaceous event. It is possible the End-Cretaceous mass extinction could be disproportionally contributing to the coefficient of association for bivalves and gastropods in our results, particularly as the End-Cretaceous event has the greatest sampling coverage of all five mass extinction events. This greater sampling coverage could also lead to higher species richness, which could potentially affect selectivity. Jablonski [1] considered the role of species richness in affecting geographical range selectivity measurements during the End-Cretaceous mass extinction, but found that it was not a factor, despite contributing to selectivity patterns during background intervals. The computational expense of including species richness per time interval in our analyses precludes the ability of assessing the role of species richness in geographical range selectivity, but it appears unlikely that species richness of genera affects clade-level selectivity during mass extinctions in this study, as Payne and Finnegan also considered species richness in their analyses and found it also was not a factor [12]. In a supplementary analysis, we consider the effect of the End-Cretaceous event by removing it for bivalves and gastropods and find that it does not significantly change the results of our study (electronic supplementary material, figure S3).

As with geographical range, extinction selectivity with respect to body size during background and mass extinction is also consistent with previous studies, despite adding geographical range as a covariable in our CMR models [10,13]. This finding demonstrates that the associations of body size and geographical range with extinction exist independently and do not arise purely through correlation with one another (e.g. size correlates with geographical range, which determines extinction probability). During background intervals, the general selectivity against smaller body size across clades could be explained by fecundity, where small ectotherms, particularly sessile forms, have lower fecundity and dispersal ability [75,76]. The variability in body size response to mass extinctions is difficult to explain because body size allometrically scales with various traits not considered here [22–24]. Moreover, because there is a general lack of support for models with multiplicative effects on extinction risk between geographical range and body size (except for rhynchonellate brachiopods for background extinction), any effect on extinction risk is not significant, particularly during mass extinctions. Notwithstanding, geographical range response to mass extinction is also variable by clade, suggesting that there might be a connected underlying intrinsic factor driving selectivity of both traits, such as physiology (e.g. [77,78]).

The overall reduction in selectivity observed during mass extinctions is primarily driven by the decrease in geographical range extinction risk. What remains unclear is why extinction selectivity becomes more variable by clade during mass extinctions relative to background intervals. The variability in intrinsic traits among clades, particularly physiology, anatomy, fecundity and generation time among other traits is expected to result in differing rates of macroevolutionary dynamics with respect to body size and geographical range, resulting in different responses to extinction and at different hierarchical levels [74]. This is particularly the case for traits that covary with other traits and each other, such as geographical range and body size. Despite the differences between geographical range and body size in terms of trait type (i.e. emergent versus organismal trait, respectively [79]), they exhibit largely shared selectivity direction among different taxonomic groups during background extinction, even if selectivity coefficients are not statistically significant (figure 2*a*). This question of whether background extinction patterns are shared across classes for many traits is open and consequential. Potentially, background extinction selectivity is similar across classes because of shared macroevolutionary trade-offs. Endemic taxa and smaller-bodied taxa have a higher probability of speciating [80–83], thus increasing their diversity, but at the cost of higher extinction risk [74]. The variability in strength and direction of selectivity observed among clades with respect to two different traits during mass extinctions suggests then, that extrinsic events briefly disrupt long-term trends driven by macroevolutionary trade-offs of intrinsic traits.

## Data Availability

Data are stored in Dryad: https://doi.org/10.5061/dryad.931zcrjqw [84]. Supplementary tables are stored in Zenodo: https://doi.org/10.5281/zenodo.8341743 [85] and R code for all analyses is also stored in Zenodo: https://doi.org/10.5281/zenodo.7816408 [86]. Supplementary material is available online [87].
